# MiRNA-296-5p promotes the sensitivity of nasopharyngeal carcinoma cells to cisplatin via targeted inhibition of STAT3/KLF4 signaling axis

**DOI:** 10.1038/s41598-024-55123-4

**Published:** 2024-03-20

**Authors:** Hai-qing Luo, Yan Wang, Jing Ren, Quan-ying Zhang, Yan Chen, Mei-hui Chen, Ning-xin Huang, Min-hua Wu, Xu-dong Tang, Xiang-yong Li

**Affiliations:** 1 Center of Oncology, the Affiliated Hospital of Guangdong Medical University, Zhanjiang, 524001 People’s Republic of China; 2 Key Laboratory for Biologically Active Molecules of Department of Education of Guangdong Province, Guangdong Medical University, Zhanjiang, 524023 People’s Republic of China; 3 Institute of Biochemistry and Molecular Biology, Guangdong Medical University, Zhanjiang, 524023 People’s Republic of China; 4 Department of Histology and Embryology, Guangdong Medical University, Zhanjiang, 524023 People’s Republic of China

**Keywords:** miRNA-296-5p, Chemosensitivity, Nasopharyngeal carcinoma, STAT3, KLF4, Biochemistry, Cancer, Molecular biology

## Abstract

Improving drug sensitivity is an important strategy in chemotherapy of cancer and accumulating evidence indicates that miRNAs are involved in the regulation of drug sensitivity, but the specific mechanism is still unclear. Our previous study has found that miR-296-5p was significantly downregulated in nasopharyngeal carcinoma (NPC). Here, we aim to explore whether miR-296-5p is involved in regulating cisplatin sensitivity in NPC by regulating STAT3/KLF4 signaling axis. The cell proliferation and clonogenic capacity of NPC cells were evaluated by CCK8 Assay and plate colony assay, respectively. The Annexin V-FITC staining kit was used to determine and quantify the apoptotic cells using flow cytometry. The drug efflux ability of NPC cells were determined by Rhodamine 123 efflux experiment. The expression of miR-296-5p, apoptosis-related genes and protein in NPC cell lines were detected by qPCR and Western blot, respectively. Animal study was used to evaluate the sensitivity of NPC cells to DDP treatment in vivo. Our results showed that elevated miR-296-5p expression obviously promoted the sensitivity of NPC cells to DDP by inhibiting cell proliferation and clonogenic capacity, and inducing apoptosis. In addition, we found that miR-296-5p inhibited the expression of STAT3 and KLF4 in NPC cells, while overexpression of exogenous STAT3 reversed miR-296-5p-mediated enhancement in cell death of DDP-treated NPC cells. In vivo studies further confirmed that miR-296-5p promotes the sensitivity of NPC cells to DDP treatment. miRNA-296-5p enhances the drug sensitivity of nasopharyngeal carcinoma cells to cisplatin via STAT3/KLF4 signaling pathway.

## Introduction

Nasopharyngeal carcinoma (NPC) is the most malignant head and neck tumors. According to the International Agency for Research on Cancer, there were about 133,354 new NPC cases diagnosed in 2020. Nevertheless, its globally geographical distribution is extremely unbalanced with more than 70% of new cases reported in east and southeast Asia, while 62,444 new cases were reported in China that accounts for 46.8% of all new NPC cases^1,2^. So far, chemo-radiotherapy has emerged as a valid tool for NPC^3^. Cisplatin (DDP) is one of the most effective chemotherapy drugs for most cancers including NPC, which inhibits tumor cell growth and induces apoptosis through the formation of cisplatin–DNA adducts^4^. However, the development of chemoresistance remains the main obstacle for an effective DDP treatment of NPC. Therefore, a comprehensive understanding of the mechanism of DDP resistance and efforts to improve its drug sensitivity is an important strategy to develop effective treatment of NPC.

MicroRNAs (miRNAs) are small and non-coding RNAs that play important roles in various biological processes of cancer cells, including proliferation, differentiation, cell cycle and apoptosis by regulating downstream target gene expression. Notably, numerous studies have found that miRNAs are involved in modulating the chemosensitivity of tumor cells^5^. For example, Wuerkenbieke et al. demonstrated that miRNA-150 downregulation contributed to the pertuzumab resistance in ovarian cancer via activating PI3K-Akt pathway^6^. Recently, Xu et al. found that miRNA-610 could affect chemoresistance to DDP in hepatocellular carcinoma through targeted silencing of the HDGF gene^7^. Moreover, miRNA-375-3p could enhance 5-fluorouracil chemosensitivity in colorectal cancer by targeting thymidylate synthase^8^. Therefore, the dysregulation of miRNAs is widely associated with tumor growth and the development of chemoresistance. Recently, we found that miRNA-296-5p was obviously downregulated and functioned as a tumor suppressor in NPC^9^. However, it remains largely unknown whether miRNA-296-5p is involved in the study of chemotherapy sensitivity in NPC.

In the present study, we found that the upregulation of miRNA-296-5p obviously promoted the sensitivity of NPC cells to DDP through inhibition of cell proliferation and clonogenic capacity and induction of apoptosis, while silencing miRNA-296-5p had the opposite effect, suggesting that miRNA-296-5p promotes CDDP chemosensitivity in NPC cells. Moreover, our results further demonstrated that overexpressing miRNA-296-5p inhibited but silencing miRNA-296-5p increased the expression of STAT3 and KLF4 in NPC cells, respectively. Mechanistically, STAT3 is the downstream target gene of the miR-296-5p, and overexpression of exogenous STAT3 counteracted miR-296-5p-mediated enhancement of DDP chemosensitivity, indicating miRNA-296-5p promotes DDP chemosensitivity by targeting STAT3/KLF4 in NPC cells. Finally, in vivo animal experiments also confirmed that miR-296-5p promoted the sensitivity of NPC cells to DDP treatment.

## Materials and methods

### Cell culture and transfection

CNE-1, CNE-2, 5–8F cells were cultured in RPMI 1640 medium supplemented with 10% fetal bovine serum (FBS) and 1% antibiotics. The cell lines were cultured at 37 °C in a humidified atmosphere with 5% CO_2_. MiR-296-5p mimic/inhibitor and the corresponding control vectors were purchased from RiboBio (Guangzhou) and transfected into NPC cells with Lipofectamine 3000 reagent following the manufactures’ protocol.

### CCK8 assay

4 × 10^3^ cells/well seeded in a 96-well plate were treated with various concentrations of DDP (0, 5, 10 and 20 μmol/L) for 48 h. The control group was treated with DMSO. At the appropriate time points, cell proliferation assay was performed by the addition of 10 μL CCK-8 solution to each well, followed by incubation at 37 °C for 1 h. Absorbance at a wavelength of 450 nm was measured using a microplate reader (BIO-RAD, USA).

### Colony assay

NPC cells were inoculated into a 35-mm cell culture dish (1 × 10^5^/dish) with 3 mL complete culture medium and cultured in a CO_2_ incubator for 24 h. On the second day, the medium was replaced with fresh medium containing different concentrations of DDP, and cells were continuously cultured for 12 days. After discarding the culture medium, cells were gently washed twice with PBS, followed by fixation with 1 mL of 4% paraformaldehyde (PFA) for 30 min at room temperature. Afterwards, cells were stained with 1% crystal violet dye solution for 30 min at room temperature. Following washing twice with PBS, the stained cells were air-dried, and then clones larger than 10 cells were observed and counted, and finally photographed under a microscope.

### Apoptosis assay

The Annexin V-FITC staining kit from Beyotime Biotechnology (Shanghai, China) was used to determine and quantify the apoptotic cells using flow cytometry according to the manufacturer’s instruction. In brief, the collected cells were suspended in the supplied binding buffer, and then stained with FITC-conjugated annexin V and PI at room temperature (RT) for 20 min in the dark. The fluorescent intensities of the cells were detected using flow cytometry, and the Annexin V^+^/PI^−^ and Annexin V^+^/PI^+^ cell populations were representative of apoptotic cells.

### Rhodamine 123 efflux experiment

Following transfection for 48 h, cells were harvested and resuspended in 1 mL complete culture medium. Verapamil hydrochloride of different volumes was added. The mixture was incubated at 37 °C with 5% CO_2_ for 30 min. At the end of incubation, 10 μL Rhodamine 123 dye was added and mixed, followed by incubation at 37 °C with 5% CO_2_ for 30 min. At the end of the incubation, cells were collected by centrifugation and then resuspended in 2 mL of complete culture medium. Then, verapamil hydrochloride of different volumes was added and mixed, followed by incubation at 37 °C with 5% CO_2_ for 30 min. Afterwards, cells were collected, resuspended and washed twice in 2 mL cold PBS by centrifugation. Finally, cells were resuspended in 1 mL cold PBS and tested in the dark. 1.0 × 10^4^ cells were collected for each tube of the sample and the fluorescence signal was determined by flow cytometry.

### Quantitative real-time PCR

Total RNA was extraction from tissue or cultured cells using RNAiso Plus (Takara, Japan), and then RNA was inversely transcribed into cDNA and used for PCR amplification. The results were analyzed by 2^−∆∆Ct^ method. The specific primers were obtained from GenCards and the primer sequences are as follows: (F) 5′-GTCAGTGGTGGACCTGACCT-3′, (R) 5′-TGCTGTAGCCAAATTCGTTG-3′ for GAPDH. (F) 5′-TGTGCGTATGGGAACACCTA-3′, (R) 5′-AGAAGGTCGTCTCCCCCTTA′ for STAT3. (F) 5′-CCCACACAGGTGAGAAACCT-3′ (R) 5′-ATGTGTAAGGCGAGGTGGTC-3′ for KLF4. (F) 5′-CAGGCCTCCCTCTCTCTTCT-3′ (R) 5′-CCAGCAGCTCCTCACACATA-3′ for Bcl-xL.

### Western blot

Western blot analysis was performed as described previously^8^. The cells were lysed in the radioimmunoprecipitation (RIPA) lysis buffer containing protease inhibitor. The protein concentrations were determined using a bicinchoninic acid (BCA; Thermo Fisher Scientific Inc) Protein Assay Kit. Equal amounts of proteins were separated through 10% sodium dodecyl sulfate–polyacrylamide gel electrophoresis (SDS-PAGE) by electrophoresis, and then samples were transferred onto a polyvinylidene fluoride (PVDF) membrane. After blocked with 5% nonfat dried milk, the PVDF membranes were immunoblotted with specific primary antibodies, respectively. Following extensively washing, the membranes were incubated with corresponding secondary antibodies conjugated with horseradish peroxidase. The immune complexes on the PVDF membrane were detected using an Electrochemiluminescence (ECL) detection system.

### In vivo experiment

BALB/c Nude mice (male, 5 weeks old, 18 ~ 22 g) were purchased from Charles River (Beijing). The protocols of our animal experiments have been approved by the Animal Care and Use Committee of Guangdong Medical University. The effect of miRNA-296-5p on NPC growth was assessed in a xenografted NPC model in BALB/c Nude mice (n = 6/group). Briefly, 0.1 mL of cell suspension of CNE-2 cells in PBS (2 × 10^5^cells/tumor) was subcutaneously injected into the right limbs of the mice. DDP/PBS was intraperitoneally administered every other day at a dose of 2 mg/kg body weight. 1 nmol of miRNA-296-5p agomir/agomir NC was injected into tumors every other day. The control group was injected with agomir NC and PBS. The major diameter (length, L) and minor diameter (width, W) were measured daily using a caliper. Tumor volume (mm^3^) = L × W^2^/2. The animals were sacrificed 17 days post-inoculation.

### Immunohistochemistry

The sections were dewaxed and hydrated, and then underwent antigenic repair using thermal repair. Then, the sections were subjected to membrane permeation and 10% goat serum sealing treatment. The primary antibody was incubated at 4 °C for 16 h. Endogenous enzyme activity was removed using hydrogen peroxide. After the secondary antibody and strept avidin–biotin Complex (SABC) incubation treatment, the protein was performed by 3,3'-diaminobenzidin (DAB) incubation. Subsequently, the nucleus was stained with hematoxylin, and the results were recorded using a light microscope.

### Statistical analysis

Three multiple holes were set for each experiment, and the experiment was repeated for 3 times for each group. SPSS 17.0 statistical software was used for statistical analysis. Measurement data were expressed as mean ± standard deviation (mean ± SD), and comparison data between the two groups were replaced by t-test after normal test and homogeneity test of variance or necessary equation substitution, and the significance level was set as 0.05.

### Ethics approval

The animal experiments were performed in accordance with the guidelines and regulations for the Care and Use of Laboratory Animals, the Animal Research: Reporting of In Vivo Experiments (ARRIVE), and approved by the Animal Center of Guangdong Medical University (GDY2003011).

## Results

### Expression of miRNA-296-5p in nasopharyngeal carcinoma

**Figure 1:**
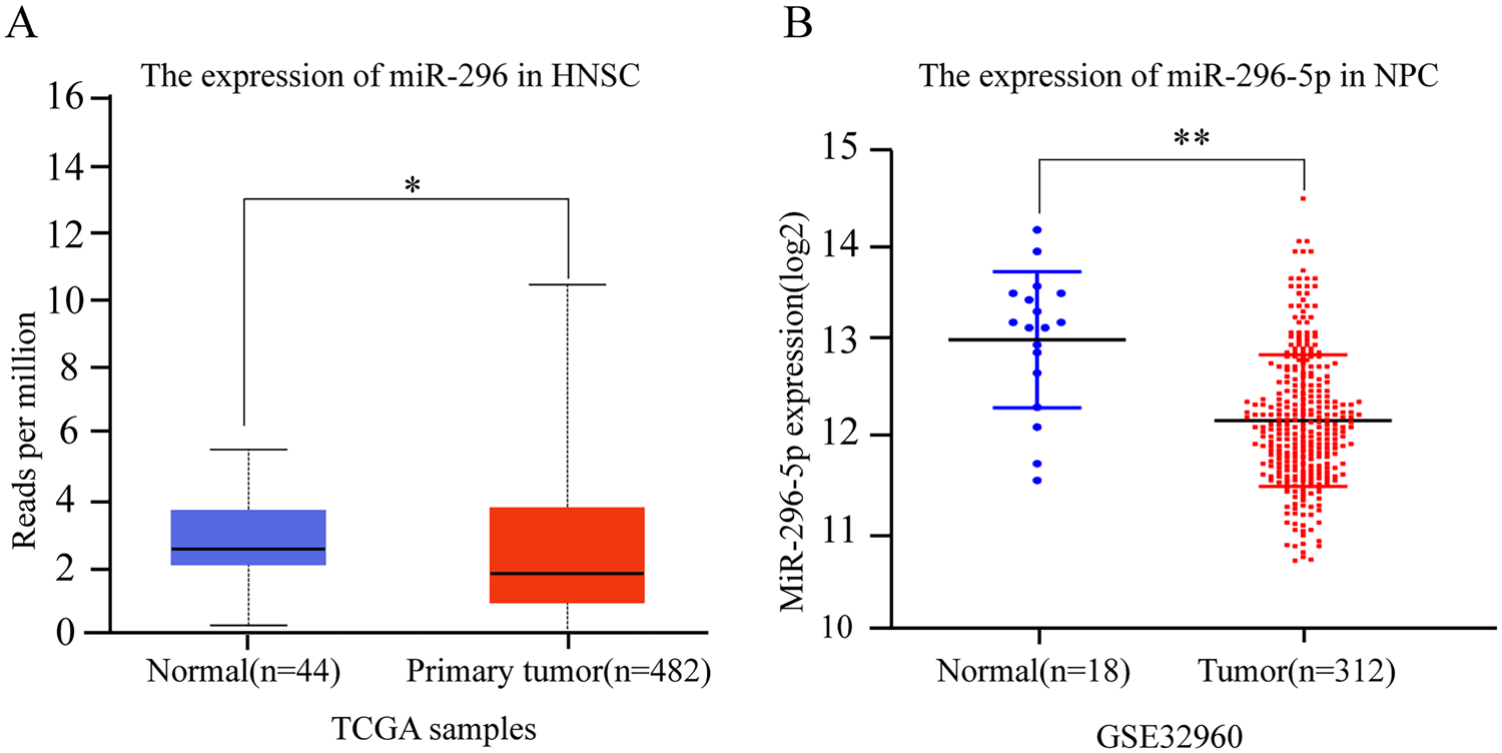
MiRNA-296-5p was downregulated in HNSCC and NPC. (**A**) The expression of miR-296-5p in HNSCC. (**B**) The expression of miR296-5p in NPC. (*p < 0.05, **p < 0.01).

To determine the expression of miRNA-296-5p in NPC, we firstly performed TCGA miRNA analysis of the dataset for a cohort of head and neck squamous cell carcinoma (HNSCC) downloaded from UALCAN^10^ (http://ualcan.path.uab.edu). As shown in Figure lA, the expression of miRNA-296 was downregulated in HNSCC compared to that in normal paracancerous tissues. In addition, we found that the expression of miRNA-296-5p was significantly downregulated in NPC compared to that in adjacent normal tissues (Fig. 1B), which is consistent with our previous results that the expression of miR-296-5p in CNE-2, CNE-1 and 5-8F cells was lower than that in NP69 cells^9^.

### MiR-296-5p effectively enhanced the antitumor effect of cisplatin (DDP) in NPC cells

**Figure 2:**
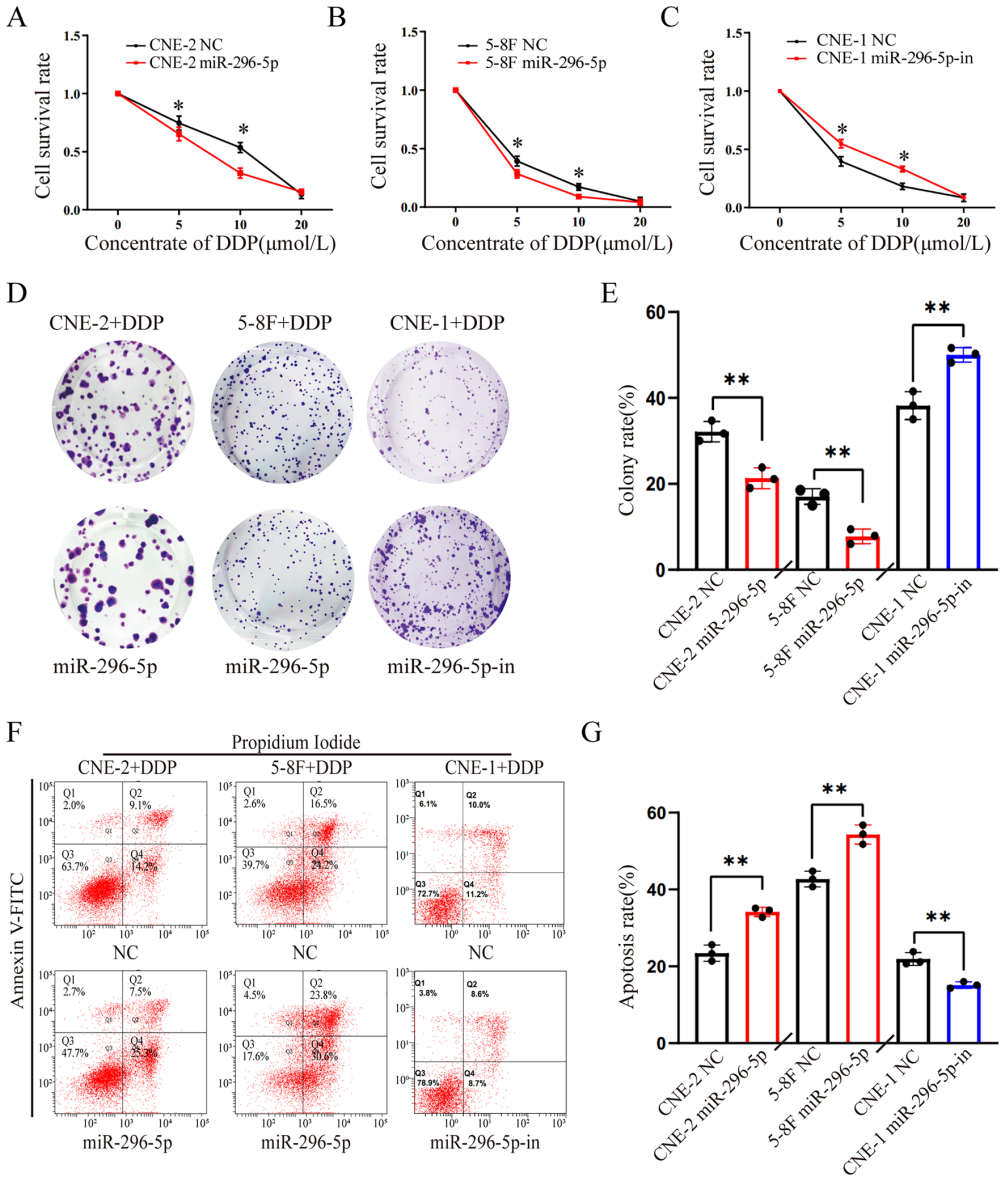
MiR-296-5p effectively enhanced the antitumor effect of cisplatin (DDP) in NPC cells. (**A**,**B**) CCK-8 assay showed that miR-296-3p mimic enhanced DDP-induced cell death in CNE-2 (**A**) and 5-8F NPC cells (**B**). (**C**) CCK-8 assay showed miR-296-3p inhibitor alleviated DDP-induced cell death in CNE-1 NPC cells. *P < 0.05 (double-tailed T test), the data represents at least three replicates. (**D**) Representative image of clonal formation of NPC cells. (**E**) Quantitation of clonal formation. *P < 0.05, the data represents at least three replicates. (**F**) Representative results of NPC cell apoptosis analyzed by flow cytometry. (**G**) Quantitation of cells apoptosis. *P < 0.05, the data represents at least three replicates. (n = 3, *p < 0.05, **p < 0.01).

To determine the role of miRNA-296-5p in regulating chemotherapy resistance of NPC cells, we firstly observed the effect of miRNA-296-5p on the viability of NPC cells treated with DDP. According to our results, up-regulation of miR-296-5p expression by transfection of NPC cells with a miR-296-5p mimic significantly enhanced cell death in both CNE-2 and 5-8F NPC cells treated with DDP at a concentration of 5 µmol/L and 10 µmol/L, respectively (Fig. 2A,B). Conversely, silencing miR-296-5p expression with a miR-296-5p inhibitor obviously alleviated DDP-induced cell death in CNE-1 NPC cells (Fig. 2C).

In addition, results from clonal formation assay also indicated that miR-296-5p enhanced DDP-induced inhibitory effect on the clonal formation ability of NPC cells, while such inhibitory effect of DDP was partially reversed by miR-296-5p inhibitor (Fig. 2D,E). Additionally, flow cytometry analysis showed that overexpression of miRNA-296-5p significantly increased DDP-induced apoptosis in CNE-2 and 5-8F cells, while down-regulation of miR-296-5p expression reduced DDP-induced apoptosis in CNE-1 cells (Fig. 2F,G. These data suggest that miR-296-5p promotes the antitumor ability of DDP in NPC cells, including inhibition of NPC cell growth and clonogenic capacity but augment of NPC apoptosis.

### MiR-296-5p inhibited drug efflux ability of NPC cells and altered the expression of the apoptosis-related genes in NPC cells

**Figure 3:**
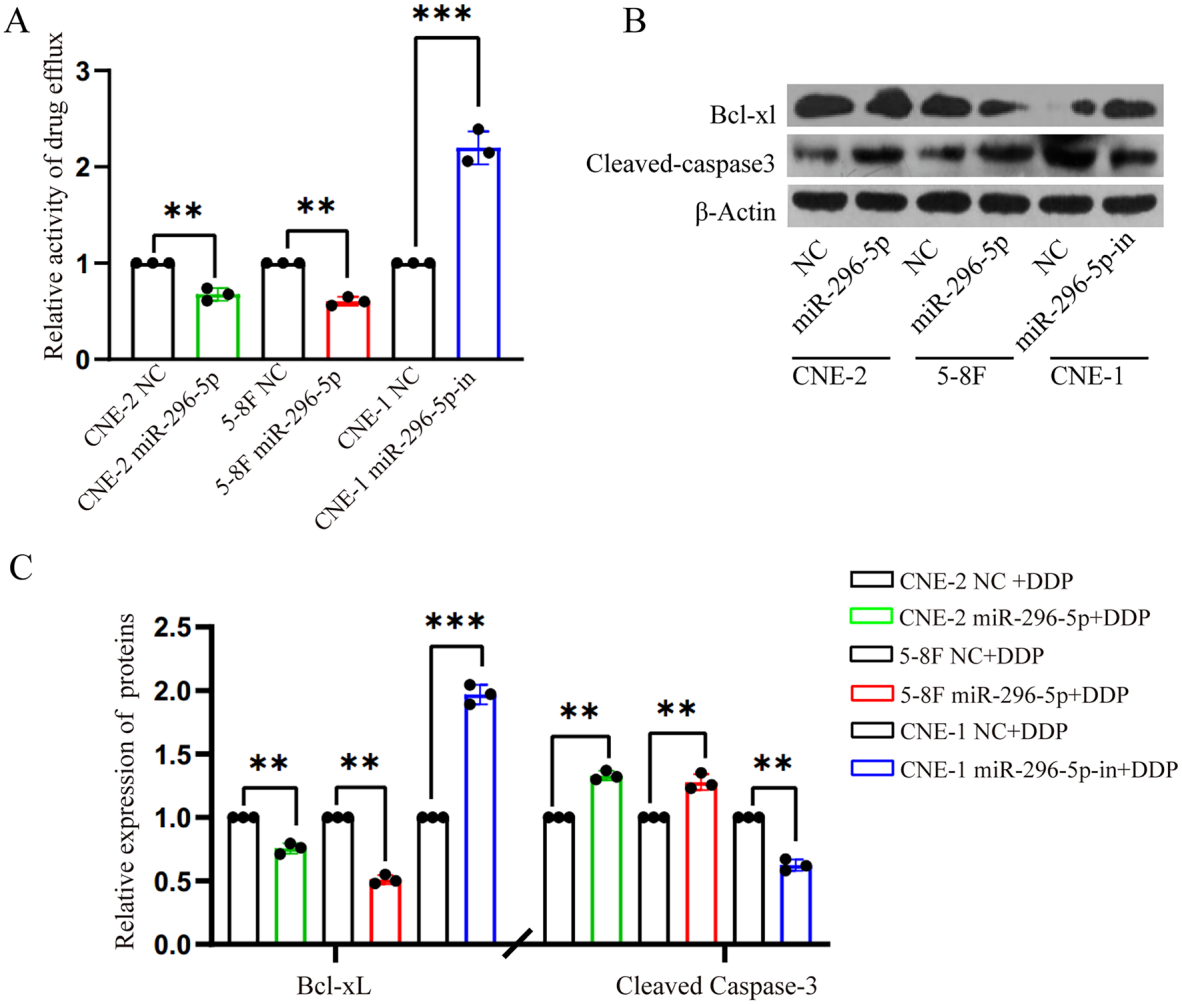
MiR-296-5p inhibited drug efflux ability of NPC cells and altered the expression of the apoptotic-related genes in NPC cells. (**A**) miR-296-5p reduced the efflux capacity of NPC cells by Rhodamine 123 efflux assays. (**B**) Western blot showed the regulatory effects of miR-296-5p on the protein expression of the apoptotic-related genes in NPC cells. (**C**) The relative expression level of apoptotic-related proteins through gray-scale analysis. *P < 0.05 (double-tailed T test), the data represents at least three replicates. (n = 3, **p < 0.01, ***p < 0.0001).

Usually, drug efflux represents one of the major mechanisms of chemoresistance of tumor cells. We then used the rhodamine 123 efflux experiment to detect the effect of miR-296-5p on drug efflux ability of NPC cells. The results showed that overexpression of miR-296-5p inhibited the drug efflux ability of NPC cells, but miR-296-5p inhibitor significantly increased the efflux ability of NPC cells (Fig. 3A). Western blot analysis showed that miRNA-296-5p mimic promoted cleaved caspase-3 expression but significantly reduced Bcl-xL expression in both CEN-2 and 5-8F cells. On the contrary, miR-296 inhibitor exerted the opposite effect on cleaved caspase 3 and Bcl-xL expressions in CNE-1 cells (Fig. 3B,C). In summary, the results suggest that miRNA-296-5p enhances the sensitivity of NPC cells to DDP-induced apoptosis, at least in part, by inhibiting the drug efflux.

### STAT3 is the target gene of miR-296-5p

**Figure 4:**
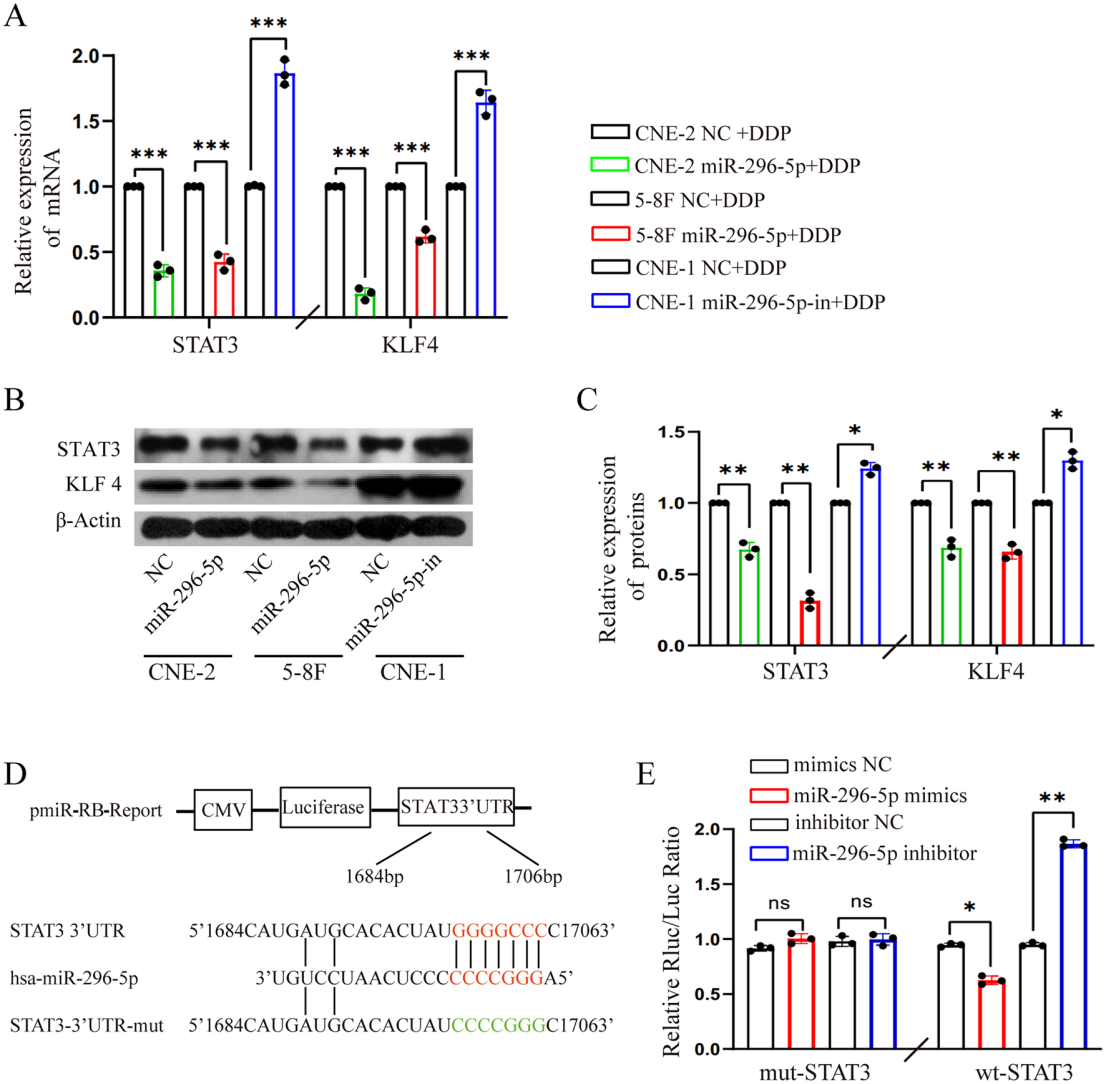
MiR-296-5p suppressed STAT3 expression by directly targeting its 3ʹ-UTR. (**A**) The mRNA expression of STAT3 and KLF4 in NPC cells treated with miR-296-5p mimics and inhibitors, respectively. (**B**) Western blot showed miR-296-5p mimics decreased while miR-296-5p inhibitors increased the protein expression of the STAT3 and KLF4 in NPC cells. (**C**) The relative expression level of STAT3 and KLF4 through gray-scale analysis. (**D**) The binding sites of miR-296-5p with wild-type or mutant STAT3 in 3ʹ-UTR (Data was taken from Targetscan). (**E**) Luciferase assays in 293 T cells co-transfected with wild-type or mutant STAT3 vectors and an miR-296-5p mimic, inhibitor or the relevant NC. *P < 0.05 (double-tailed T test), the data represents at least three replicates. (n = 3, ns: p > 0.05, *p < 0.05, **p < 0.01, ***p < 0.0001).

To clarify the underlying mechanism by which miR-296-5p enhances DDP sensitivity in NPC cells, TargetScan was used to find the potential target genes of miR-296-5p, among which we chose to verify is *STAT3*, a gene closely related to drug resistance. Quantitative real-time PCR and western blot analysis showed that STAT3 protein expression was significantly decreased in CNE-2 and 5-8F NPC cells transfected with miR-296-5p mimics but significantly increased in CNE-1 cells transfected with the miR-296-5p inhibitor (Fig. 4A–C). Interestingly, we also found that the expression of Kruppel-like factor 4 (KLF4), a zinc finger-containing transcription factor, was similarly inversely regulated by the expression of miR-296-5p (Fig. 4A–C).

Then the luciferase assays were performed to determine whether STAT3 is a downstream target gene of miR-296-5p. Briefly, we constructed pmiR-RB-REPORT luciferase reporter plasmids containing regions of the 3ʹ-UTR of STAT3 which were subsequently co-transfected with the miR-296-5p mimics, miR-296-5p inhibitor, or the corresponding negative controls into 293 T cells (Fig. 4D). Our results showed that the miR-296-5p mimics largely decreased the luciferase activity of the STAT3 reporter plasmid, while the miR-296-5p inhibitor elevated the luciferase activity. However, the miR-296-5p mimics and miR296-5p inhibitor exerted no obvious effect on the luciferase activity of the mut-STAT3 reporter plasmid (Fig. 4E). These results support the hypothesis that miR-296-5p negatively regulates STAT3 expression in NPC cells via directly binding to its 3ʹ-UTR.

### STAT3 overexpression rescues the miR-296-5p-imediated enhanced DDP sensitivity in NPC cells

**Figure 5:**
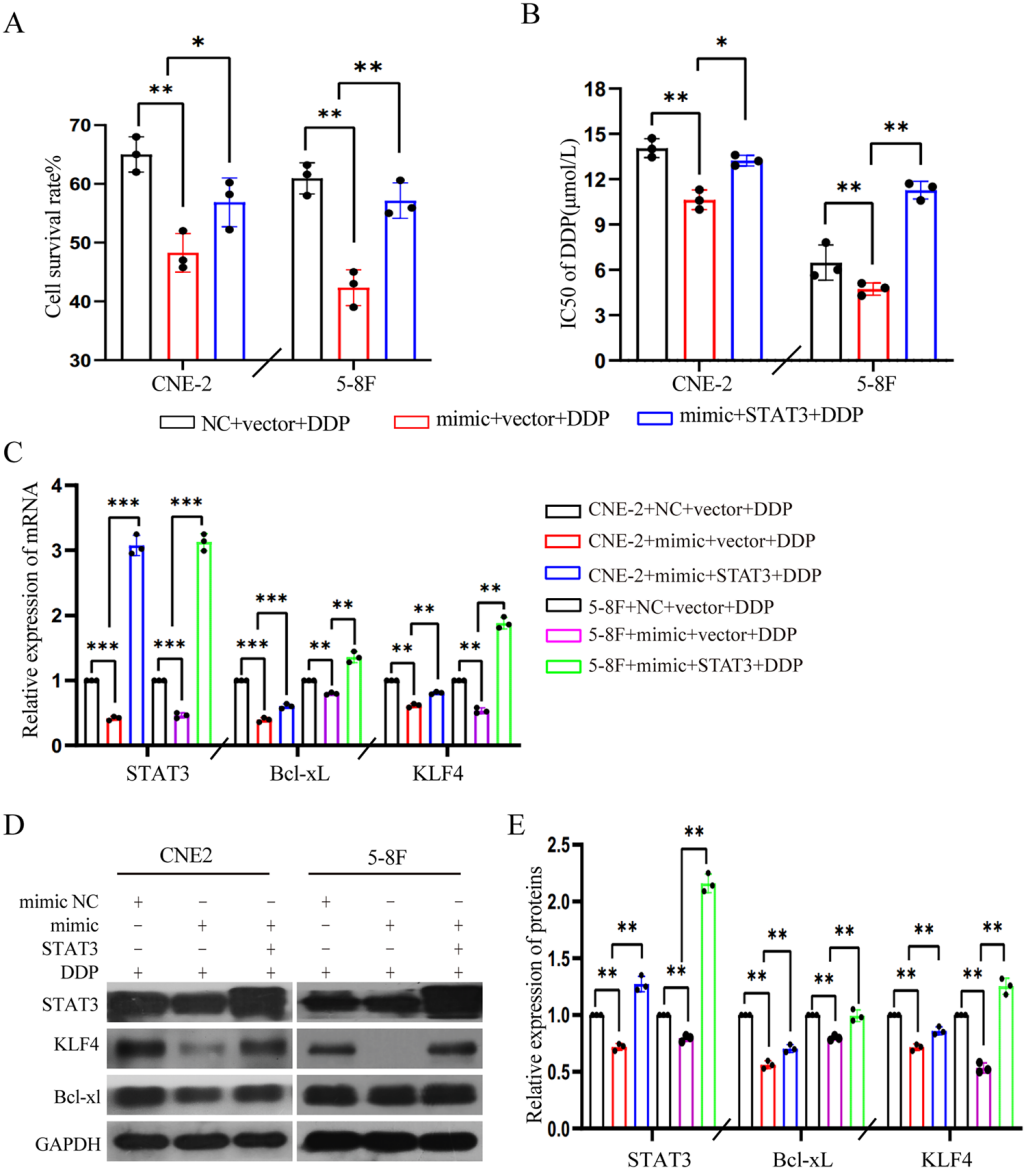
STAT3 overexpression rescued miR-296-5p-mediated enhanced DDP sensitivity in NPC cells. (**A**) CCK8 assays showed exogenous STAT3 reversed miR-296-5p-mediated enhanced DDP sensitivity in NPC cells. (**B**) The IC50 of miR-296-5p on DDP sensitivity with or without exogenous STAT3 in NPC cells. (**C**) Exogenous STAT3 rescued miR-296-5p mediated downregulation of KLF4 and Bcl-xL mRNA expressions in NPC cells by qPCR. (**D**) Western blotting confirmed exogenous STAT3 rescued miR-296-5p-mediated downregulation of KLF4 and Bcl-xL protein expressions in NPC cells. (**E**) The relative expression level of STAT3, KLF4, and Bcl-xL proteins through gray-scale analysis. *P < 0.05; **P < 0.01; ***P < 0.001 (double-tailed T test), the data represents at least three replicates. (n = 3, *p < 0.05, **p < 0.01).

Since miR-296-5p enhanced DDP sensitivity in NPC cells by down-regulating STAT3 expression, we investigated whether such effects could be rescued by exogenous STAT3. To this end, CNE-2 and 5-8F cells were co-transfected with miR-296-5p mimics and exogenous STAT3, followed by treatment with DDP. Cell viability assay indicated that exogenous STAT3 counteracted miR-296-5p-mediated enhanced DDP sensitivity in NPC cells (Fig. 5A,B). Accordingly, Quantitative real-time PCR and western blot analysis revealed that exogenous STAT3 attenuated miR-296-5p-mediated down-regulation of KLF4 and Bcl-xl in NPC cells (Fig. 5C–E). These data strongly support that miRNA-296-5p promotes DDP sensitivity in NPC cells via targeted inhibition of STAT3/KLF4 signaling axis.

### MiRNA-296-5p enhanced the sensitivity of NPC cells to DDP treatment in vivo

**Figure 6:**
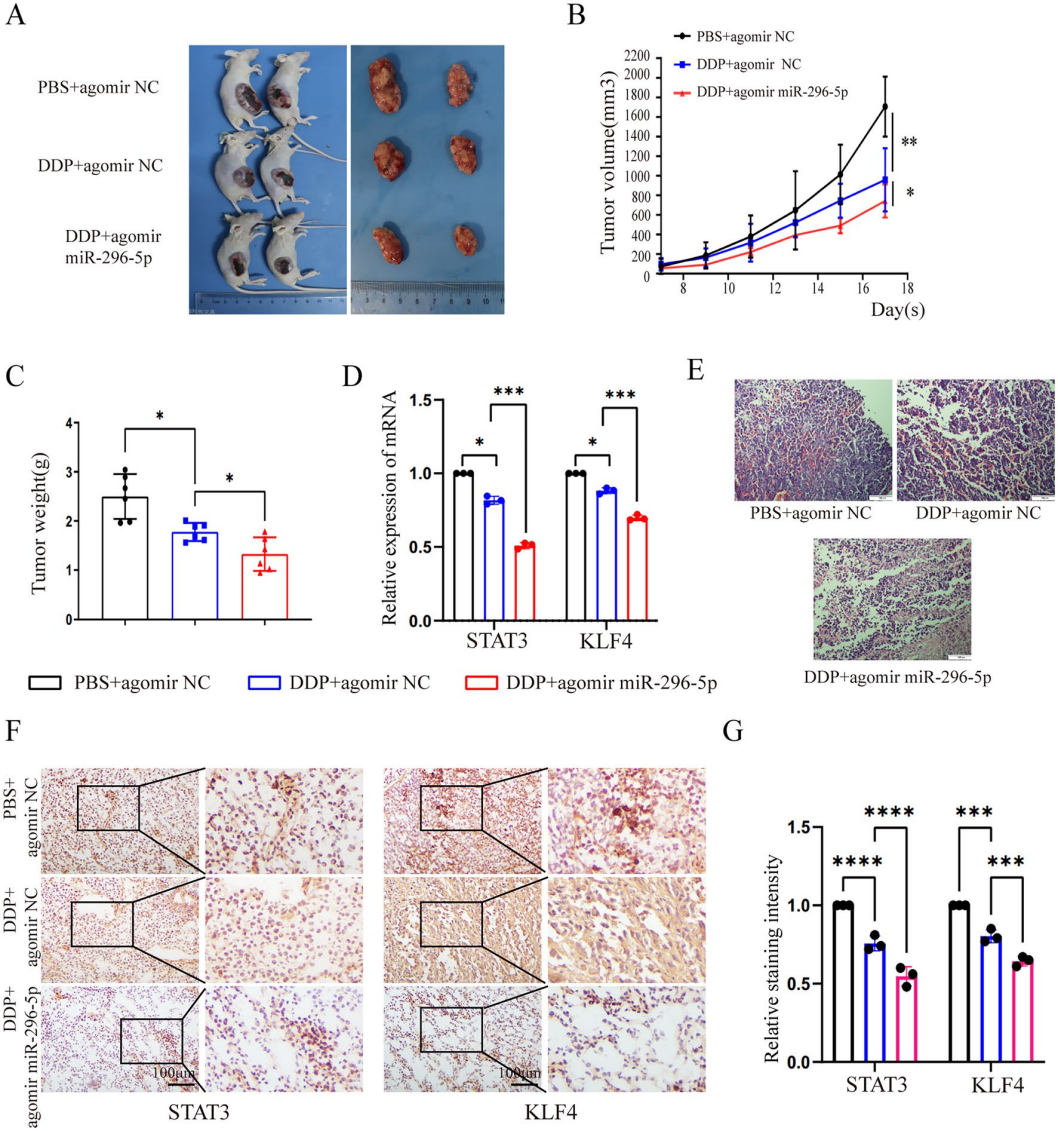
MiRNA-296-5p enhanced the drug sensitivity of CNE2 cells to DDP in vivo. (**A**) Representative image of the xenografted CNE2 tumors in Balb/c nude mice (n = 6/group). (**B**) The survival curve was based on tumor volumes (n = 6/group). (**C**) The tumor weight of each group (n = 6/group). (**D**) The mRNA expression of STAT3 and KLF4 in xenograft NPC tumors by qPCR. (**E**) H&E stained tissue of tumors. *P < 0.05; **P < 0.01 compared with control. (n = 3, *p < 0.05, **p < 0.01, ***p < 0.0001).

We then evaluated the important role of miR-296-5p in NPC chemoresistance in xenografted CNE2 tumor model BALB/c Nude mice. 7 days after inoculation of CNE2 cells, we observed obvious formation of solid tumors in all mice. Then the mice were randomly divided into three groups (PBS + agomir NC, DDP + agomir NC, DDP + agomir miR-296-5p, n = 6/group). A dose of 1 nmol of the miRNA-296-5p agomir was injected into tumors every other day. DDP/PBS was intraperitoneally injected at a dose of 2 mg/kg every other day. The tumor sizes were measured and recorded every other day. After 10 days, a remarkable difference in the tumor size was observed (Fig. 6A). Both the volume and weight of tumors from the miR-296-5p agomir + DDP group were less than the other two groups (Fig. 6B,C). Interestingly, our results of qPCR showed that miR-296-5p agomir significantly inhibited the mRNA levels and protein expression of STAT3 and KLF4 in xenografted tumor tissues (Fig. 6D,F,G), which is consistent with the results of in vitro cell experiments, suggesting that miR-296-5p could enhance the sensitivity of nasopharyngeal carcinoma to cisplatin by downregulating STAT3 and KLF4 expression, and further promote tumor cell death. Finally, our results of H&E staining confirmed the xenograft NPC tumors as solid tumors (Fig. 6E). Collectively, these results demonstrated that miR-296-5p also enhanced the sensitivity of NPC cells to DDP treatment in vivo.

## Discussion

At present, radiotherapy is still the first choice for the treatment of early NPC, but chemotherapy or chemotherapy combined with radiotherapy has been shown to be an effective treatment modality for NPC patients, including those with a late-stage and recurrent NPC^11^. Cisplatin, a kind of metal platinum complex drugs, has potent anti-tumor effect by destroying cell DNA and inhibiting cell mitosis, which has become one of the most commonly used drugs for combined chemotherapy for various types of malignant tumors, including NPC. However, the development of chemoresistance has become one of the major hurdles for the wide use of cisplatin as a chemotherapeutic drug in clinic^12^. Therefore, it is of great practical significance to clarify the mechanism of cisplatin resistance and to enhance the sensitivity of NPC cells to this drug so as to improve its therapeutic efficacy in NPC patients.

It is generally believed that miRNAs are not only closely related to the occurrence and development of diverse cancers, but also contributes to the development of chemoresistance of cancer cells to various therapeutic drugs^13,14^. A growing number of studies have found that miRNAs can regulate the chemosensitivity of tumor cells^14,15^, and combination of miRNAs with chemotherapy is expected to become a new paradigm for cancer therapy. Regarding the role of miR-296-5p in cancer, Shivapurkar et al. found a low expression level of miR-296-5p in cervical cancer, and the decrease in circulating miR-296-5p was associated with shorter survival and poor response to treatment with Sunitinib and Capecitabine^16,17^. However, another study revealed that the expression of miR-296-5p was obviously increased in glioblastoma, while downregulation of miR-296-5p increased the sensitivity of esophageal cancer cells to both P-glycoprotein-related and P-glycoprotein-nonrelated drugs^18^. These findings have implied that the differential expression and biological functions of miR-296-5p are tumor context-dependent.

According to our previous studies, miR-296-5p was significantly downregulated in NPC and served as a tumor suppressor to negatively regulate the cell growth and metastasis of NPC cells^9^. In the present study, we demonstrated that miRNA-296-5p remarkably enhanced DDP-induced apoptosis and inhibitory effect on the clonal formation ability of NPC cells. Meanwhile, miRNA-296-5p decreased the expression of *Bcl-xL*, an anti-apoptotic gene, but concomitantly increased the expression of the cleaved caspase-3, a pro-apoptotic gene, in NPC cells. In contrast, downregulation of miRNA-296-5p expression in NPC cells resulted in the opposite effect on the expression of these apoptosis-related genes. These findings indicate that miRNA-296-5p could increase the sensitivity of NPC cells to cisplatin.

Signal Transducer and Activator of Transcription 3 (STAT3) has been shown to play an important role in tumorigenesis and progression by regulating cell proliferation, differentiation, metastasis and apoptosis^19–21^, which might be one of the main targets for tumor therapy^22–24^. Increased STAT3 activation (p-STAT3) has been not only implicated in driving NPC progression and metastasis but also be clinically associated with the advanced stages (stage III or IV) of NPC^25^. Interestingly, several studies have reported that STAT3 is frequently associated with Klf4 expression^26,27^. In addition, Klf4 has been shown to be a downstream target of STAT3^28^, and STAT3 plays a key role in regulating properties and functions of cancer stem cells^30^. Interestingly, a recent study showed that miR-196-5p promoted stemness of colorectal cancer cells by activating STAT3 signaling pathway^29^. KLF4, a key factor in pluripotency transcriptional network, not only plays a pivotal role not only in regulating various cellular processes such as cell cycle, apoptosis, and metabolism but also in maintaining the self-renewal capacity of stem cells, including cancer stem cells (CSCs)^31,32^. Recently, Wei et al. reported that KLF4-induced CSCs contributes to therapeutic resistance in breast cancer^33^. In the present study, we found that miR-296-5p not only enhanced the sensitivity of NPC cells to DDP, but also effectively inhibited the expression of STAT3 and KLF4, suggesting that miRNA-296-5p enhances DDP sensitivity of NPC cells possibly through the inhibition of STAT3 / KLF4.

## Conclusions

In summary, the present study reveals that miRNA-296-5p enhances the chemosensitivity of NPC cells to cisplatin via interfering with STAT3/KLF4 signaling pathway. The elucidation of the mechanisms underlying the interaction between miR-296-5p and DDP chemosensitivity in NPC cells has shed light on the development of novel therapeutic approaches for NPC.

### Supplementary Information

Supplementary Information.

## Data Availability

The datasets analyzed in Fig. 1 are available in the TCGA and GEO repository as follows: TCGA data: https://xenabrowser.net/datapages/?dataset=TCGA-HNSC.mirna.tsv&host=https%3A%2F%2Fgdc.xenahubs.net&removeHub=https%3A%2F%2Fxena.treehouse.gi.ucsc.edu%3A443. GEO data: https://www.ncbi.nlm.nih.gov/geo/query/acc.cgi?acc = GSE32960.

## Abbreviations

DDP: Cisplatin
NPC: Nasopharyngeal carcinoma
miRNAs: MicroRNAs
CSCs: Cancer stem cells
STAT3: Signal transducer and activator of transcription 3
PFA: Paraformaldehyde
SDS-PAGE: Sodium dodecyl sulfate–polyacrylamide gel electrophoresis
PVDF: Polyvinylidene fluoride
ECL: Electrochemiluminescence
HNSCC: Head and neck squamous cell carcinoma
