# Supplementary figures and images for: MiRNA-296-5p promotes the sensitivity of nasopharyngeal carcinoma cells to cisplatin via targeted inhibition of STAT3/KLF4 signaling axis
Source: Sci Rep. 2024 Mar 20;14:6681. doi: 10.1038/s41598-024-55123-4 (PMC10954770; doi:10.1038/s41598-024-55123-4)

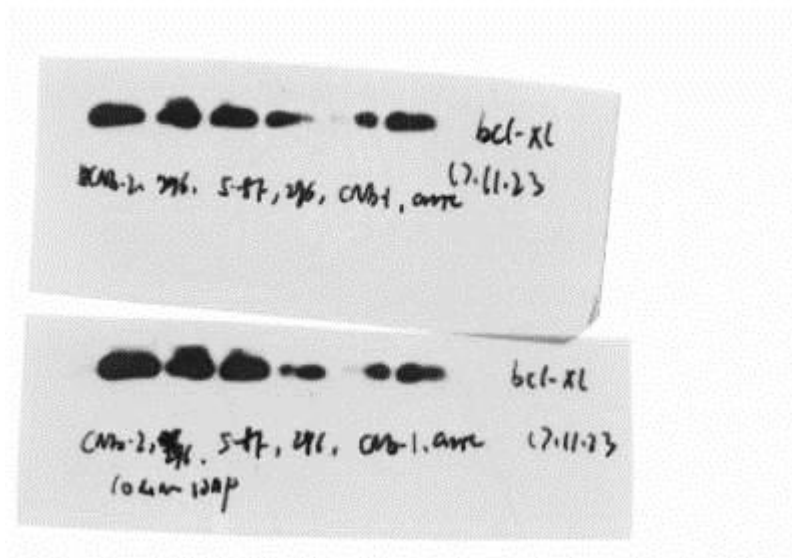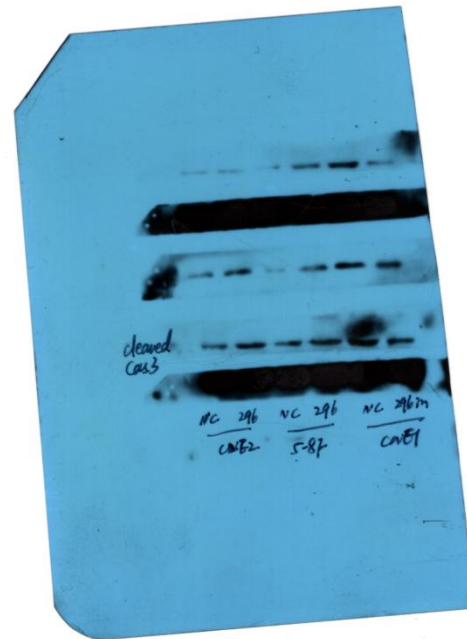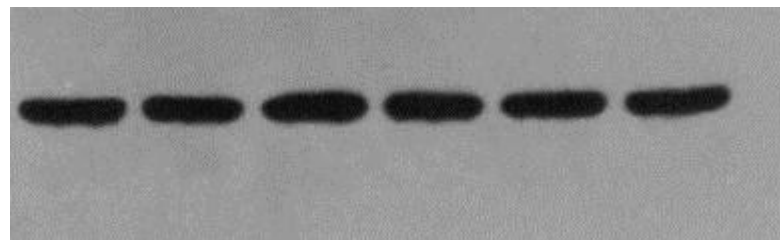

Fig.5b

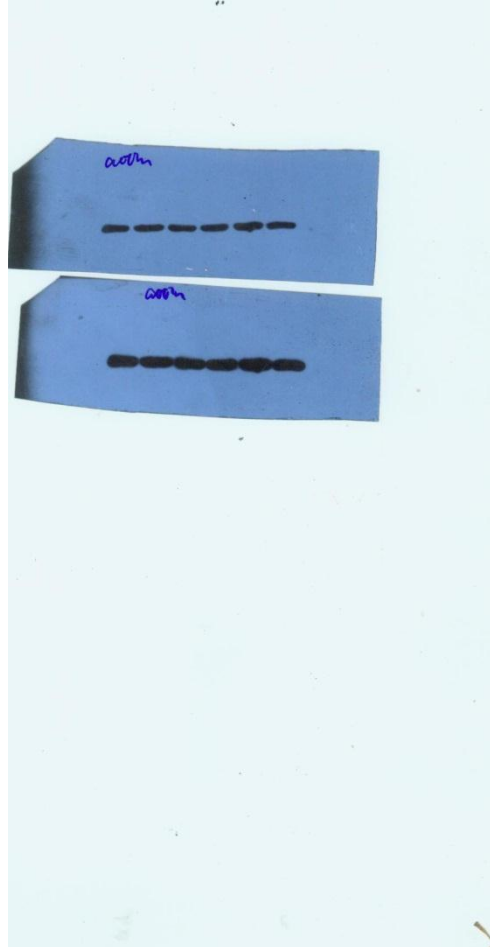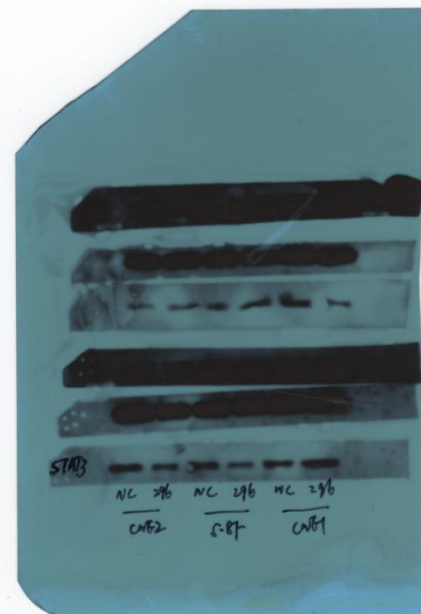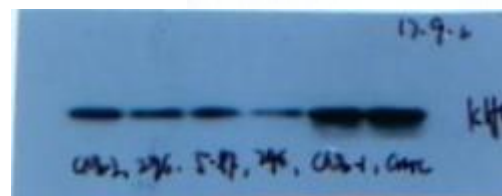

Fig.6a



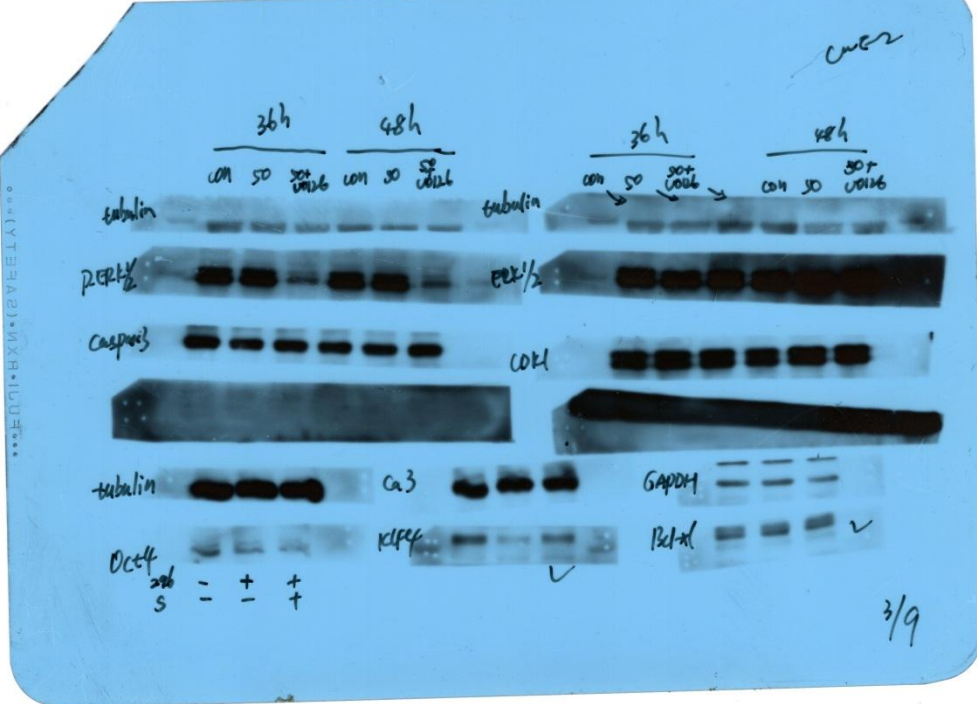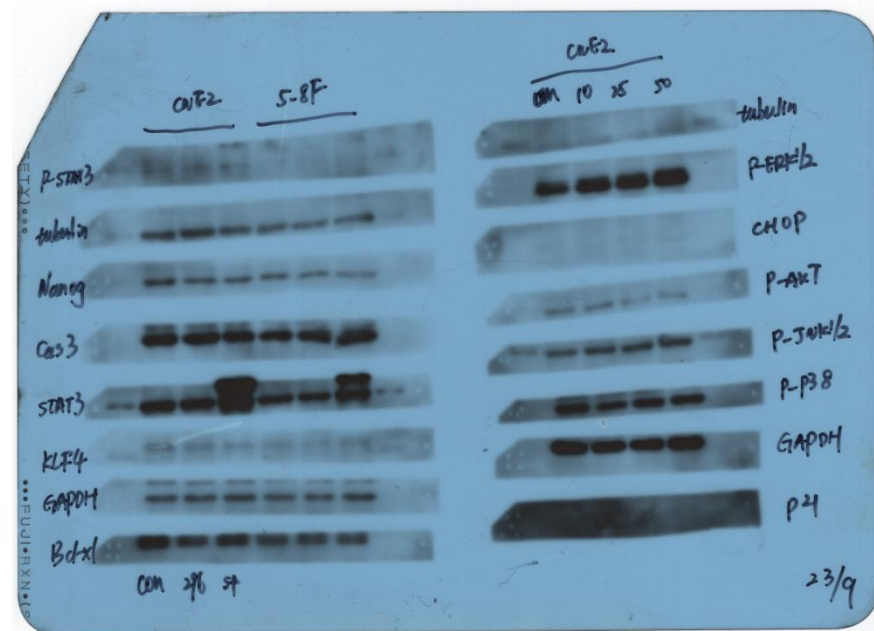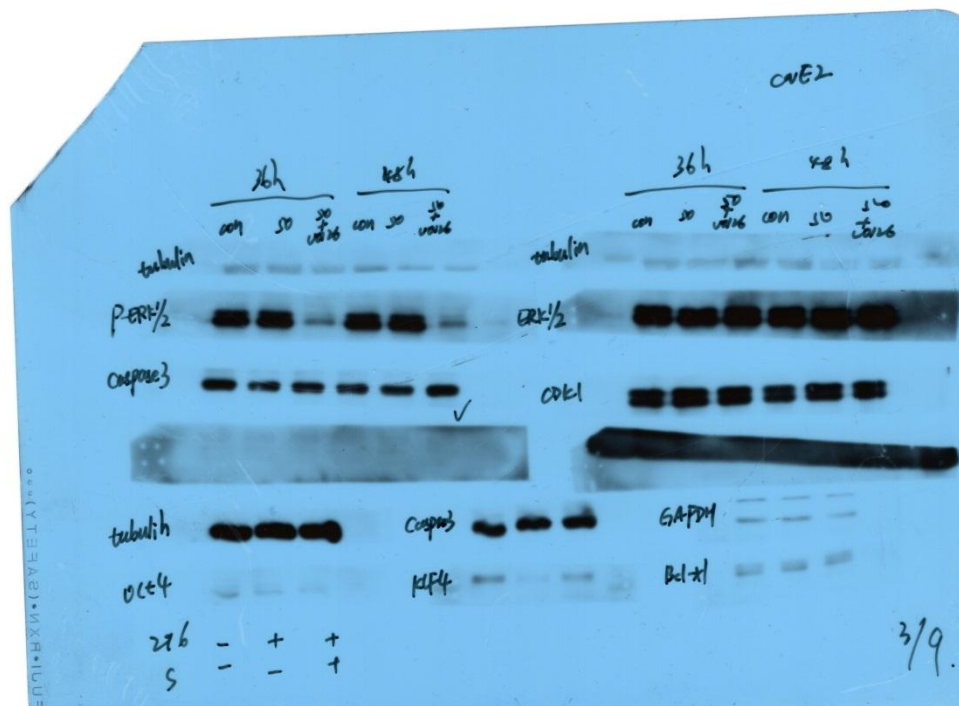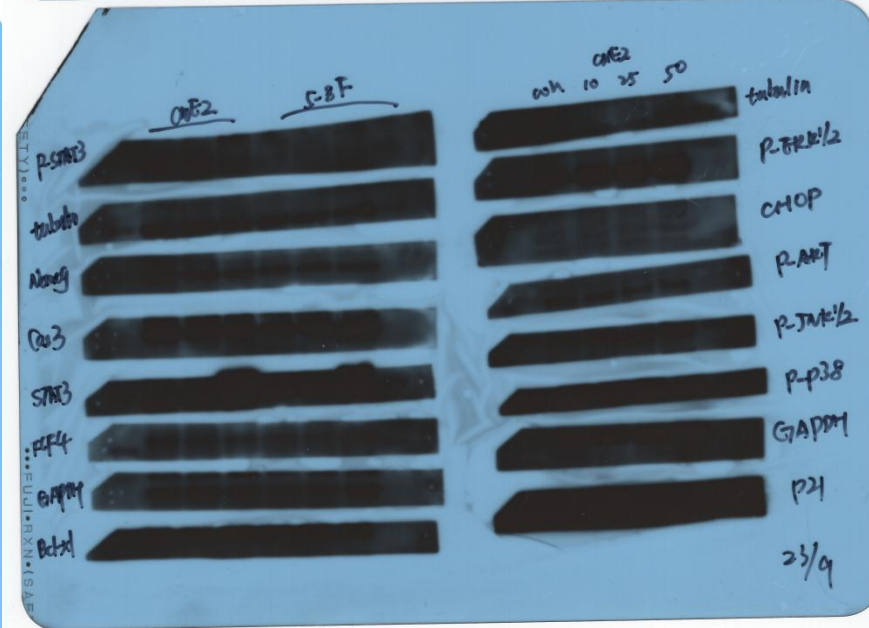

Fig.7c-2

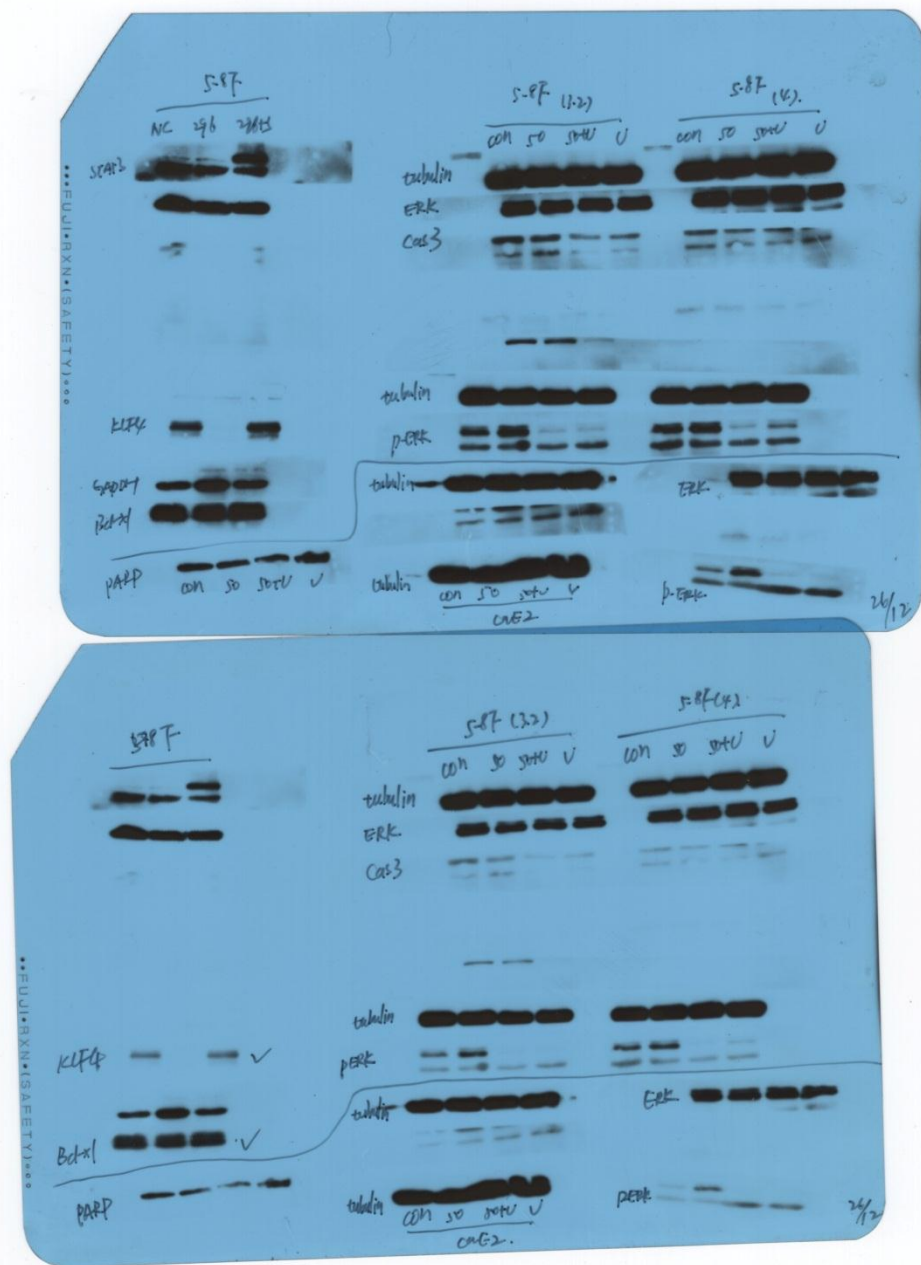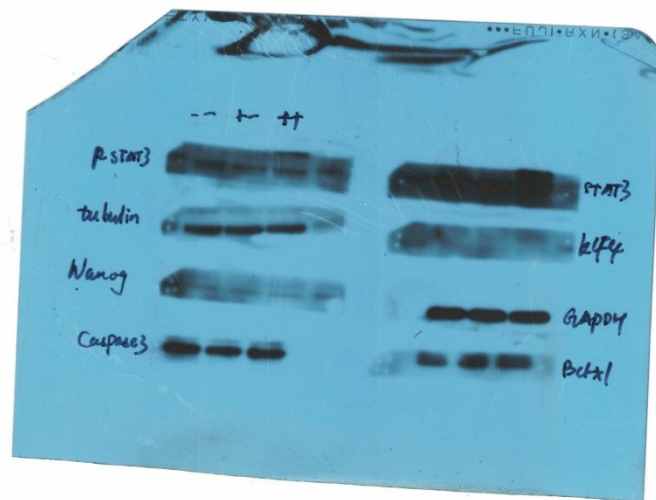

Fig.7c-3

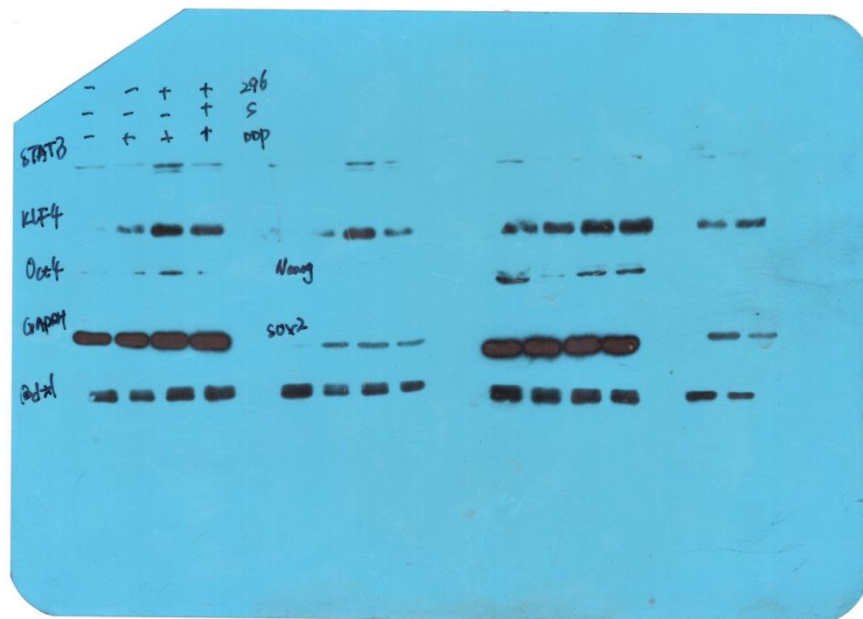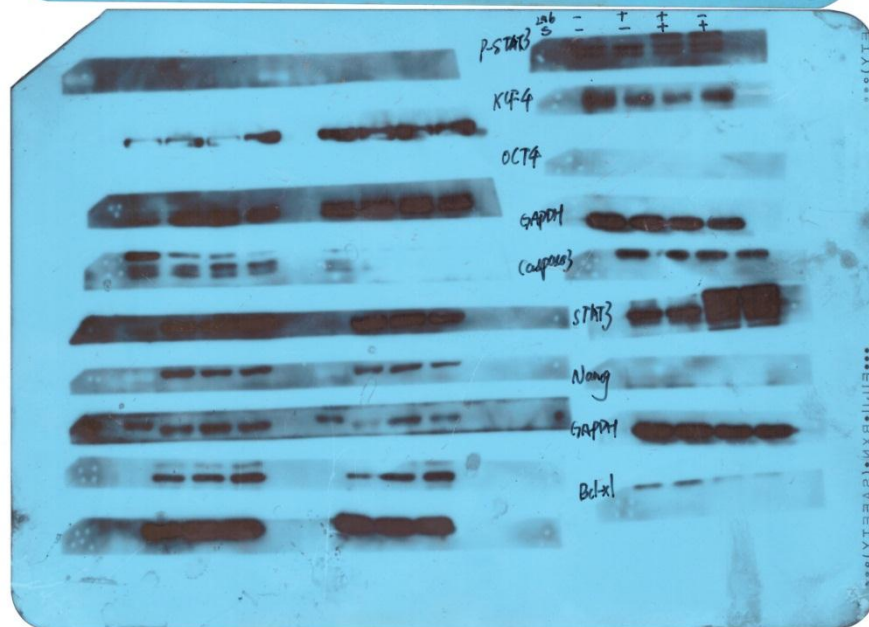

Fig.7c-4

Supplement: Supplementary file 1 — Supplementary Information. [file 41598_2024_55123_MOESM1_ESM.pdf]
